# Descriptive Study about Bodyweight Status of Extremadura Adolescents. Are We Applying the Best Indicator as the Reference Parameter?

**DOI:** 10.3390/biology10070662

**Published:** 2021-07-14

**Authors:** María Mendoza-Muñoz, Laura Muñoz-Bermejo, Rafael Gómez-Galán, Violeta Calle-Guisado, Raquel Pastor-Cisneros, Miguel Ángel Garcia-Gordillo, José Carmelo Adsuar, Jorge Carlos-Vivas

**Affiliations:** 1 Promoting a Healthy Society Research Group (PHeSO), Faculty of Sport Sciences, University of Extremadura, 10003 Cáceres, Spain; mamendozam@unex.es (M.M.-M.); violetacg@unex.es (V.C.-G.); raquelpc@unex.es (R.P.-C.); jadssal@unex.es (J.C.A.); jorgecv@unex.es (J.C.-V.); 2 Social Impact and Innovation in Health (InHEALTH), University of Extremadura, 10003 Cáceres, Spain; rgomez@unex.es; 3 Facultad de Administración y Negocios, Universidad Autónoma de Chile, Sede Talca 3467987, Chile; miguel.garcia@uautonoma.cl

**Keywords:** adolescent, anthropometric, BMI, childhood obesity, classification systems, percentiles, youth

## Abstract

**Simple Summary:**

There are no agreed criteria that establish childhood obesity thresholds, based on BMI, which may be used to diagnose overweight/obesity in adolescents. This tool has been determined as the most practical and least costly method in classifying bodyweight status in adolescents. However, it is an indicator of bodyweight, and not fat mass. This study assessed and compared bodyweight status of Extremadura adolescents by sex and age using international (World Health Organization), national (Faustino Obergozo Foundation), and regional references (Extremadura). A single-measure descriptive study (bodyweight and height) was conducted in 4130 adolescents aged between 12–17 years. BMI was calculated to classify participants in the bodyweight categories. The WHO criteria classified 542 individuals in a different category compared to FO, and 1028 individuals with respect to EX. Moreover, FO classified 684 adolescents in a different category than EX. Despite the concordance in diagnostic overweight/obesity observed when comparing the three classifications, differences in percentage of participants for each category were found in all pairwise comparisons (WHO–FO, WHO–EX, and FO–EX). Thus, WHO, FO, and EX criteria differ when estimating overweight/obesity prevalence in adolescents, being able to fall into a wrong diagnosis of overweight or obese.

**Abstract:**

*Background:* There is no agreed criteria that establishes childhood obesity thresholds based on BMI, which may be used to assess adolescent overweight/obesity. This tool has been determined at the most practical and least costly in classifying bodyweight status in adolescents. However, it is an indicator of bodyweight and not adiposity. *Aims:* To assess bodyweight status of Extremadura adolescents by sex and age using international, national, and regional reference criteria and comparing the different diagnoses criteria. *Methods*: A descriptive cross-sectional study was conducted with 4130 adolescents (12–17 years). Bodyweight and height were assessed. *Results*: Pairwise comparisons indicates that the World Health Organization (WHO) classified 542 individuals in a different category compared to Faustino Obergozo (FO), and 1028 individuals with respect to the Extremadura adolescents’ percentiles (EX). Moreover, FO classified 684 adolescents in a different category than EX. Despite the concordance in diagnostic criteria (by Cohen’s kappa test) reported between the WHO, FO, and EX for all bodyweight categories in both sexes and all age ranges, significant differences were found (assessed by Cochran Q test and McNemar test as post-hoc) between the WHO and FO for all bodyweight proportion except in the thinness category in girls (15–17 years) and boys (12–14 years). Meaningful differences were also obtained comparing WHO and EX for each bodyweight category in all ages and sexes. Comparisons between FO and EX revealed significant differences for all bodyweight categories in all participants except for overweight in girls (12–14 years) and boys (15–17 years) and normal weight and obesity in girls (15–17 years). *Conclusions*: the WHO, FO, and EX criteria present different outcomes estimating overweight and/or obesity prevalence in adolescents aged between 12 and 17 years. The change from Extremadura criteria to the WHO reference will result in more adolescents being diagnosed as overweight or obese.

## 1. Introduction

The World Health Organization (WHO) defines overweight and obesity as a chronic disease characterized by abnormal or excessive body fat accumulation that represents a risk for health [1]. More specifically, childhood obesity has increased ten-fold in the last 40 years, estimating that there were around 124 million obese children in 2016 [1]. Thus, it constitutes one of the primary challenges and public health issues in advanced societies [2]. Unfortunately, this disease goes unnoticed in many cases, leading to numerous comorbidities during childhood [3]. Its relevance lies in its persistence during adolescence and adulthood [4]. It is estimated that about 55% and 70% of global obese children and adolescents, respectively, will be obese as adults [5].

Spain is considered one of the countries that presents the highest overweight and obesity incidence in the world [6]. Several studies have explored overweight and obesity levels in Spanish child and youth populations [7,8,9,10]. At the regional level, few studies have reported specific data on the prevalence of overweight and obesity in the Autonomous Community of Extremadura. In 2017, the *Encuesta Nacional de Salud Española* (ENSE) [11] showed that the percentage of overweight and obesity in Extremadura children and adolescents aged among 2 and 17 years old was 11.88% and 10.38%, respectively. However, this study did not specify the percentage or sample size from Extremadura. Other studies on the prevalence of overweight and obesity that have considered the community of Extremadura have also failed to report region-specific data [7,8,9,10].

The appropriate interpretation of anthropometric measurements depends on a large extent on the use of appropriate growth curves to compare and interpret anthropometric values [12]. This tool has been determined, albeit with limitations, to be the most practical and least costly means of classifying body mass index (BMI) in children and adolescents [13].

Focusing on childhood obesity, there are no agreed criteria that establish the threshold, based on BMI, which may be used to learn if a child or adolescent presents overweight or obesity [14]. Numerous studies and research have recommended BMI as an indicator to assess for overweight and obesity in children and adolescents [15,16] due to its positive correlation with adiposity previously found for this population [17,18,19]. However, it should be noted that BMI represents both fat mass and fat-free mass, being an indicator of bodyweight and not adiposity [20]. Thus, the two most widely used and internationally relevant criteria for establishing bodyweight categories in relation to BMI are those established by the WHO [6] and the International Obesity Task Force (IOTF). On one hand, the WHO states that the bodyweight status of every child or adolescent will be established according to the following thresholds based on BMI standard deviation (SD): (1) < −3 SD, “thinness”; (2) < −2 SD, “normal weight”; (3) > + 1 SD, “overweight”; and (4) > + 2 SD, “obese”. On the other hand, the IOTF [21] uses BMI cut-off points to establish sex-specific overweight or obesity in children based on the reference population. Nevertheless, in Spain, the percentile growth tables and graphs published by the Faustino Orbegozo Eizaguirre Foundation, since its first edition in 1985, have been taken as the reference [22]. More specifically, the health records of children from Extremadura are based on the tables and graphs produced and published in 2004, derived from the Growth Curves and Tables Study (longitudinal and cross-sectional studies) [23]. These tables are stratified by age and sex, and consider as cut-off points the values corresponding to the 85th and 95th percentiles for overweight and obesity, respectively [14]. Thus, the fact that there is no single criterion or reference value may suppose a limitation in interpreting the data. In fact, the prevalence of overweight and obesity slightly fluctuates depending on the different criteria used [24,25]. The hypothesis deduces that bodyweight status will vary according to the reference values used, which might generate disparate diagnoses of this issue in the focus population.

Therefore, this study aimed to assess the bodyweight status of Extremadura adolescents aged between 12 and 17 years, according to sex and age using international, national, and regional reference criteria, to analyze if differences exist in diagnoses when comparing the international, national, and regional reference criteria.

## 2. Materials and Methods

### 2.1. Study Design

A descriptive cross-sectional study was carried out during a 14-month period. Multistage stratified sampling was used for the study. The units of the successive stages were provinces, typology of place (urban or rural), school, academic year, sex and pupils (surveyed persons). Surveys were conducted in a total of 40 secondary schools.

### 2.2. Sample Size

Data obtained from the National Statistical Institute of Spain showed that the population for age ranging between 12 and 17 years in Extremadura was 74,239 people (51.3% males, 48.7% females). Considering these 74,239 individuals and assuming a 99.9% certainty, a precision of 2%, and an expected proportion of 50%, a sample size of 3928 adolescents would be enough to conduct the present study. These computations were conducted using the following equation:

$$n=\frac{N*Z_{a}^{2}*\;p*q}{d^{2}*\left(N-1\right)+Z_{a}^{2}*p*q}$$

where *N* is the total population; *Z_a_^2^* = 3.29 (if 99,9% certainty); *p* = expected proportion (in this case 5% = 0.05); *q* = 1 − *p* (in this case 1 − 0.5 = 0.5); and *d* = precision (in this case we want 2%).

Specifically, a sample of 4130 individuals from that age range participated in this study to obtain reliable estimates of the survey at the autonomous community level. Every stratum was separated into sections with probability proportional to its size. Proportional assignment among health care areas strata were used. Thus, within each stratum, the size of the rural or urban area (substratum) was applied. Individuals were randomly selected within the educational center of the aimed populations with proportional type sampling in age and sex.

### 2.3. Ethics Approval

The Bioethics and Biosafety Committee at the University of Extremadura approved the present study procedures, according to the guidelines of the Declaration of Helsinki (reference code 11/2006). All participants and their parents or legal guardians signed an inform consent form accepting their participation in the study.

### 2.4. Participants

Forty secondary schools from Extremadura (Spain) participated in this study. Schools were randomly chosen to ensure the selection of a representative sample from all Extremadura health care areas. A total of 4130 adolescents (2128 males and 2002 females), aged between 12 to 17 [14.25 (1.48)] years were assessed. Participants needed to meet the following eligibility criteria: (1) aged between 12 and 17 years; (2) living in the Autonomous Community of Extremadura; (3) be authorized by their parents or legal guardians; and (4) accept participation in the study.

### 2.5. Procedures and Measures

All data were obtained by qualified health personnel in the corresponding schools. The measurements were taken under standardized conditions according to the protocols described in the Data Collection Procedure Manual, specifically developed for the Childhood Obesity Surveillance Initiative (COSI) [6]. To measure height and bodyweight, study participants were asked to take off their shoes and remove their socks as well as any heavy clothing or accessories. Height measurement was performed using a stadiometer (Tanita Tantois, Tanita Corporation, Tokyo, Japan) on a vertical surface, ensuring the perpendicular position of measurement scale. For the measurement, participants stood up with straight shoulders and relaxed arms. Results are expressed in centimeters to the nearest millimeter. A scale (HD 380 BK Digitale noire, Tanita Corporation, Tokyo, Japan) was used to evaluated bodyweight in kg, up to the nearest 100 g. The following equation was used to calculate the participants’ BMI: bodyweight (kg) divided by height squared (m^2^). Each participant underwent a single assessment of height and bodyweight.

### 2.6. Statistical Analyses

Statistical analyses were carried out using SPSS (Version 25, IBM SPSS, Armonk, NY, USA) software and personal data were kept anonymous. Data are presented as number of participants and percentage based on the total sample, stratified by sex and age range. The age range was established considering the categories previously proposed by Sawyer et al. [26], who differentiated between teenagers (~12–14 years old) and older adolescents (~15–17 years old).

Data normality and homogeneity were tested using the Kolmogorov–Smirnov and Levene’s tests, respectively. Frequencies of bodyweight status were determined using the cut-offs of three classification systems: the WHO, Faustino Obergozo Foundation (FO), and Extremadura percentiles (EX). Bodyweight categories for the WHO classification were established following its growth standards based on BMI standard deviation criteria, as [27]: (1) < −3 SD, “thinness”; (2) < −2 SD, “normal weight”; (3) > +1 SD, “overweight”; and (4) > +2 SD, “obese”. For the FO classification, Spanish growth charts of the Foundation were used [23], and the categories were classified as: percentile ≤ 3, “thinness”; percentiles > 3 to < 85, “normal weight”; percentile ≥ 85 to < 97, “overweight”; and percentile ≥ 97, “obesity”. Likewise, bodyweight categories for EX classification were fixed according to EX percentiles, following the same thresholds than FO.

Then, the Mann–Whitney U test was applied to check sex and age range differences for bodyweight, height, and BMI. Multiple-comparisons between the different classifications in prevalence were analyzed applying the Cochran Q test. Then, the McNemar test was conducted as post-hoc to obtain pairwise comparisons outcomes. Significant differences were considered for *p* ≤ 0.05. Between-classification concordance level or agreement was also analyzed by applying Cohen’s kappa test. Kappa thresholds were established following the classification of Landis and Koch [28]: <0.00, “poor”; 0.00 to 0.20, “light”; 0.21 to 0.40, “fair”; 0.41 to 0.60, “moderate”; 0.61 to 0.80, “substantial”; and 0.81 to 1, “almost perfect”.

## 3. Results

Table 1 shows the main characteristics for all participants stratified by sex. A total of 4130 adolescents aged between 12 and 17 years old formed the final sample. For between-sex comparisons, there were significant differences for bodyweight and height, which were significantly higher in boys than girls.

Table 2 displays the main characteristics for all participants and stratified by sex and age range. Results showed significant differences between the 12–14 and 15–17 age groups for bodyweight, height, and BMI, these variables being higher for the older age range in both boys and girls. Between-sex comparisons for the 12–14 age group revealed that bodyweight and height were significantly higher in boys than girls. For the 15–17 age group, in addition to bodyweight and height, a significant greater BMI was found in boys compared to girls.

Table 3 shows the pairwise comparisons between WHO, FO, and EX criteria on the number of participants classified in the same or different bodyweight categories. Results indicate that the WHO and FO criteria classified 3588 adolescents into the same category; however, the WHO classified 107 and 435 individuals in the lower and upper category, respectively, with respect to FO. Likewise, the comparison between the WHO and EX criteria revealed that both classifications rated 3102 participants in the same category; however, the WHO classified 6 and 1022 individuals in the lower and upper category, respectively, in relation to EX. Finally, FO and EX criteria graded 3428 participants into the same category; however, FO classified 16 and 668 adolescents in the lower and upper category, respectively, than EX. Moreover, FO rated 18 participants in the two upper categories compared to EX.

Table 4 shows the concordance in diagnostic criteria between the WHO, FO, and EX references according to bodyweight category, age and sex.

Results showed a fair to moderate agreement (k = 0.532 to 0.254; *p* < 0.001) between FO and EX references for the obesity category. However, there was no agreement of the WHO with the FO and EX diagnosis criteria for this bodyweight category. A light to fair agreement was observed between the WHO and EX in 12–14 year old girls (k = 0.344 to 0.147; *p* < 0.001) and 15–17 year old boys (k = 0.247 to 0.186; *p* < 0.001). Similarly, the agreement between the three references criteria was light to fair (k = 0.352 to −0.128; *p* < 0.001) for boys aged between 12 and 14 years. Moreover, there was fair agreement in the thinness category between the WHO and FO criteria (k = 0.249; *p* < 0.001) and light between the FO and EX criteria (k = 0.093; *p* < 0.001) in boys aged among 15 and 17. The remaining pairwise comparisons between the three diagnosis criteria were moderate to perfect for all bodyweight categories in both sexes and all age ranges.

The prevalence of bodyweight status is presented by age group and sex in Table 5. There was a significant difference between the WHO and the FO references for all proportion except in the thinness category in girls between 15 to 17 years (1% vs. 3.5%) (*p* = 0.065) and in the thinness category in boys between 12 to 14 (0.9% vs. 0.6%) (*p* = 0.125). Significant differences between the WHO and EX references were obtained for each weight category for all age and sex groups. For the FO and EX comparison, significant differences were found for all weight categories in all participant groups except to the overweight category in girls between 12 to 14 years (12.7% vs. 13.5%) (*p* = 0.631) and for boys between 15 to 17 (10.6% vs. 12.8%) (*p*= 0.103) and for the normal weight (82.5% vs. 83.2%) (*p* = 0.451) and obesity (7.7% vs. 2.4%) (*p* = 0.418) category for girls between 15 to 17 years.

## 4. Discussion

The main finding of this study showed that the bodyweight status of adolescents varies according to the reference criteria used (the WHO, Faustino Orbegozo Foundation, and reference values obtained from Extremadura). Thus, it should be highlighted that the original sample characteristics used for growth percentile computations plays a fundamental role in the classification of bodyweight status. For instance, a change on reference criteria from Extremadura to the WHO would result in more adolescents being diagnosed as overweight or obese.

The pairwise comparisons between the WHO, FO, and EX criteria on the number of participants classified in the same or different bodyweight categories showed that 542 (13.1%) adolescents were classified in different categories when using the WHO and FO criteria, 1028 (24.8%) for the WHO and EX criteria, and 702 (17%) for the FO and EX criteria. Therefore, the modality of classification could lead to a different diagnosis (overweight, obese, etc.) based on the use of the diverse growth chart.

Moderate to perfect agreement was obtained for all bodyweight status categories with some relevant exceptions: fair agreement for 15–17 years children in the WHO–FO and FO–EX comparisons for the thinness category; slight agreement in the WHO–EX for both age ranges in girls and 12–14 years boys for overweight; slight agreement in WHO-FO and FO-EX comparatives for boys aged 12–14 years also in overweight category; a modest agreement in FO–EX comparative; and no agreement in the WHO–FO and WHO–EX comparisons for the obesity category.

The moderate to excellent agreement obtained in some categories is unusual when comparing national and international references values [29,30,31]. However, similar values have been found for the obesity category using two different reference systems: the WHO and the Centers for Disease Control and Prevention (CDC) [32].

In our study, the concordance rates between the three reference systems were generally high (with the exceptions mentioned). However, there were significant differences between the bodyweight status categories of the three reference systems.

Regarding thinness status, there were significant differences between the three reference systems except for the youngest boys and girls aged 12–14 years for the WHO–FO comparative. For normal weight, there were also significant differences between the three reference systems, except for 15–17 year old girls in the FO–EX comparison. Concerning the overweight category, there were also significant differences between the three systems except for 12–14 year old girls and 15–17 year old boys in FO-EX comparative. Finally, in the obesity category, significant differences were observed between the three systems except for girls aged between 15 and 17 years in the FO–EX comparison.

Consequently, there were significant differences for all categories of bodyweight status, age, and sex of adolescents when comparing the WHO and EX criteria. The FO–EX comparison showed the least differences found, which may be due to both reference systems having been developed in Spain with samples of children and adolescents from the same country but from different regions that present differences. Along this line, the ALADINO 2011 study indicated that there were differences in the prevalence in overweight between different regions of Spain. A lower prevalence of overweight has been indicated for boys in Madrid, Catalonia, Aragon, Basque Country, La Rioja, and Castilla y León, while a higher proportion of overweight was found in Galicia, the Balearic Islands, and Extremadura. Likewise, in females, the lowest frequency of overweight have been found in Madrid, Catalonia, Aragon, Basque Country and Cantabria, while the highest were in Castilla y León, Navarra, and the Balearic Islands [10]. Recent studies on overweight and obesity prevalence in Spain [9,33] follow the WHO criteria. The PASOS [9] and *Estudio Nutricional de la Población Española* (ENPE) [33] studies showed 34.9% and 34% prevalence, respectively, similar to our results using the WHO criteria. Thus, this prevalence could change if other criteria are used, since other studies in Spain have shown that there are differences in prevalence depending on the use of one criterion or another [34].

If the WHO classification is taken as a reference, the proportions of “overweight” adolescents would increase, and the percentages in the “obese” category would be increased in boys. These results agree with previous studies, where the WHO criteria informed a higher prevalence of overweight and obesity in comparison to other national or international references values [30,31,35,36,37,38,39], which could overestimate overweight and obesity in the Extremadura context. According to our data, 1274 children would be diagnosed as overweight or obese in Extremadura using the WHO criteria, while only 621 children would be classified into these categories if Extremadura percentiles criteria are applied, or 891 in the case of the FO criteria. This means that a height difference in the diagnostics of overweight and obesity in adolescents depends on the reference used.

In summary, the findings from this study could have an impact on public health policy guidelines. If the WHO criteria are used, diagnoses and interventions would be aimed at controlling overweight and obesity in adolescents who, assessed with the Extremadura references, would be classified as normal weight [35]. The same could occur if the FO criteria are taken as the references since females could be diagnosed as obese, while they would not be classified so considering the Extremadura percentiles. In relation to this, it also leads us to the debate on the use of the oldest or the most current growth charts, as the acceleration of height and bodyweight in Spain is evident, if we compare data from 2008 [40] with studies 20 years ago [23,41,42,43]. An adolescent classified as overweight with the reference values of 20 years ago could be classified as normal weight today. Thus, an erroneous diagnosis could have important implications for clinical management strategies and interventions in public health and education.

Therefore, the fact of not having a single criterion or reference value constitutes a limitation when interpreting the results obtained in the different research studies, as previous studies have already reported [24,25]. Thus, the prevalence of overweight and obesity slightly fluctuates depending on the different criteria applied. Hence, it is necessary to know the tables used to be able to contextualize the results. In other words, it is essential that overweight and obesity prevalence data are always accompanied by the measurement criteria or reference tables used. Moreover, a comparison between the prevalence assessed with two different measurement criteria should be taken with special caution.

Nevertheless, this study presents some limitations. First, the use of BMI instead of body composition measurements, which would allow a better diagnosis of bodyweight status. BMI does not discriminate between fat mass and fat-free mass; thus, it is an indicator of bodyweight, but not adiposity [44,45,46]. Moreover, BMI can be affected by variations in body water content, bone mass and muscle mass and may misclassify fat mass, especially in children and adolescents with large muscle development [47,48]. Thus, some individuals may have high BMI values, corresponding to overweight and obese conditions, showing low values of fat-free mass and high values of fat mass. However, individuals with high fat-free mass and low fat mass may also present a high BMI, erroneously falling into the overweight and obese categories. In fact, these individuals may have muscle hypertrophy, which is common in male adolescents [49]. Therefore, considering all the above, it would be interesting to study an alternative or complementary indicator that does not present such limitation by using the combined use of bodyweight and height with other measures such as perimeters or skinfolds. Other limitations are the possible bias that may cause the cross-sectional study design since each participant is once assessed. It could be interesting to conduct similar studies performing the follow-up of participants along the full adolescence stage. Finally, the low generalizability of results obtained might also constitute a limitation because the sample was selected from only one region of Spain. It would be important that future studies are conducted including a worldwide representative population to expand the possibility of generalizing the results and exploring new reference indicators that can be applied and generalized to worldwide adolescent populations (such as fat mass, perimeters, skinfolds, ethnicity, sociocultural status…).

## 5. Conclusions

Based on our results, the WHO and EX criteria differently categorized for each bodyweight status in all age and sex groups. Likewise, the FO and EX criteria also classified participants in different categories except for overweight in females between 12 to 14 years and males between 15 to 17 and in normal weight and obesity for females between 15 to 17 years.

The reference tables developed by the WHO, Faustino Orbegozo Foundation, and Extremadura used to classify bodyweight status are based on BMI. The tables generally presented different results in terms of estimating overweight and/or obesity prevalence in adolescents aged from 12 to 17 years old. The change from the Extremadura reference to the WHO reference will result in more adolescents being diagnosed as overweight or obese. Thus, it would be necessary to unify a criterion adapted to every population, stabilizing a new indicator that could be the combined use of bodyweight and height with other measures such as perimeters or skinfolds.

## Figures and Tables

**Table 1:** Characteristic and anthropometry of participants segmented by age group and sex.

	Boys	Girls	*p*
N (%)	2128 (51.5)	2002 (48.5)	
	**Mean** (**SD**)	**Mean** (**SD**)	
**Age** (years)	14.26 (1.48)	14.25 (1.48)	0.885
**Bodyweight** (kg)	59.03 (13.96)	54.22 (10.22)	<0.001
**Height** (m)	1.65 (0.10)	1.60 (0.07)	<0.001
**BMI** (kg·m^−2^)	21.44 (3.76)	21.18 (3.52)	0.090

*p*-values from the Mann–Whitney U test.

**Table 2:** Characteristic and anthropometry of participants segmented by age group and sex.

	Boys		Girls	
	12–14	15–17	12–14	15–17
N (%)	1160 (28.03)	968 (23.38)	1091 (26.36)	911 (22.01)
	**Mean** (**SD**)	**Mean** (**SD**)	**Mean** (**SD**)	**Mean** (**SD**)
**Age** (years)	13.09 (0.79)	15.65 (0.68)	13.08 (0.79)	15.65 (0.66)
**Bodyweight** (kg)	53.89 (12.77)	65.32 (12.71) ^c^	52.07 (9.99) ^a^	56.80 (9.89) ^b,d^
**Height** (m)	1.60 (0.09)	1.71 (0.08) ^c^	1.58 (0.06) ^a^	1.61 (0.06) ^b,d^
**BMI** (kg·m^−2^)	20.77 (3.74)	22.24 (3.62) ^c^	20.72 (3.52)	21.72 (3.44) ^b,d^

^a^ Significant difference between 12–14 girls and boys for *p* ≤ 0.05. ^b^ Significant difference between 15–17 girls and boys for *p* ≤ 0.05. ^c^ Significant difference between 12–14 and 15–17 boys for *p* ≤ 0.05. ^d^ Significant difference between 12–14 and 15–17 girls for *p* ≤ 0.05.

**Table 3:** Number of participants who have been classified in the same category and in a different category between the WHO, FO, and EX classification.

WHO-FO			WHO-EX			FO-EX			
−1	0	+1	−1	0	+1	−1	0	+1	+2
N (%)	N (%)	N (%)	N (%)	N (%)	N (%)	N (%)	N (%)	N (%)	N (%)
107 (2.6)	3588 (86.9)	435 (10.5)	6 (0.1)	3102 (75.1)	1022 (24.7)	16 (0.4)	3428 (83)	668 (16.2)	18 (0.4)

Zero indicates that participants have been rated in the same category for both ratings: a positive value indicates that the first classification grading in a upper level than the second; a negative value indicates that the first classification rating in a lower level than the second.

**Table 4:** Concordance in diagnostic criteria by the WHO, FO, and EX references according to bodyweight category, age, and sex group.

		N	WHO–FO	WHO–EX	FO–EX
**Girls**	**12–14**	Thinness	1 ^a^	0.534 ^a^	0.534 ^a^
		Normal weight	0.906 ^a^	0.588 ^a^	0.669 ^a^
		Overweight	0.680 ^a^	0.344 ^a^	0.147 ^a^
		Obesity	−0.017	0.448 ^a^	−0.013
	**15–17**	Thinness	0.554 ^a^	0.430 ^a^	0.659 ^a^
		Normal weight	0.802 ^a^	0.659 ^a^	0.830 ^a^
		Overweight	0.656 ^a^	0.483 ^a^	0.513 ^a^
		Obesity	−0.017	0.532 ^a^	−0.021
**Boys**	**12–14**	Thinness	0.776 ^a^	0.566 ^a^	0.446 ^a^
		Normal weight	0.595 ^a^	0.415 ^a^	0.750 ^a^
		Overweight	0.352 ^a^	−0.128 ^a^	0.281 ^a^
		Obesity	−0.012	0.254 ^a^	−0.010
	**15–17**	Thinness	0.249 ^a^	0.513 ^a^	0.093 ^a^
		Normal weight	0.755 ^a^	0.559 ^a^	0.654 ^a^
		Overweight	0.574 ^a^	0.186 ^a^	0.247 ^a^
		Obesity	−0.002	0.381 ^a^	−0.002

WHO: World Health Organization criteria; FO: Faustino Obergozo Foundation percentiles; EX: Extremadura percentiles; ^a^ Kappa coefficient significant *p* < 0.001.

**Table 5:** Prevalence of different bodyweight categories based on thee WHO, FO, and EX by sex and age range and pairwise comparison between them.

		N	WHO (%)	FO (%)	EX (%)	WHO-FO	WHO-EX	FO-EX
**Girls**	**12–14**	Thinness	0.9	0.9	2.5	1	<0.001 *	<0.001 *
		Normal weight	69.2	73.1	81.7	<0.001 *	<0.001 *	<0.001 *
		Overweight	22.1	12.7	13.5	<0.001 *	<0.001 *	0.631
		Obesity	7.8	13.3	2.4	<0.001 *	<0.001 *	0.011 *
	**15–17**	Thinness	1.0	1.8	3.5	0.065	<0.001 *	<0.001 *
		Normal weight	78.3	82.5	83.2	<0.001 *	<0.001 *	0.451
		Overweight	15.1	8.0	10.9	<0.001 *	<0.001 *	0.004 *
		Obesity	5.6	7.7	2.4	<0.001 *	<0.001 *	0.418
**Boys**	**12–14**	Thinness	0.9	0.6	2.1	0.125	0.001 *	<0.001 *
		Normal weight	59.3	77.5	82.6	<0.001 *	<0.001 *	<0.001 *
		Overweight	25.7	10.9	13.0	<0.001 *	<0.001 *	0.083
		Obesity	14.1	10.9	2.3	<0.001 *	<0.001 *	0.001 *
	**15–17**	Thinness	0.7	0.1	2.1	0.031 *	<0.001 *	<0.001 *
		Normal weight	68.5	78.2	82.5	<0.001 *	<0.001 *	<0.001 *
		Overweight	20.7	10.6	12.8	<0.001 *	<0.001 *	0.103
		Obesity	10.1	11.1	2.6	<0.001 *	<0.001 *	<0.001 *

WHO: World Health Organization criteria; FO: Faustino Obergozo Foundation criteria; EX: Reference values from the Extremadura population. * Significant differences for *p* ≤ 0.05.

## Data Availability

The datasets used during the current study are available from the corresponding author on reasonable request.
